# Novel Candidate Genes Involved in an Initial Stage of White Striping Development in Broiler Chickens

**DOI:** 10.3390/ani14162379

**Published:** 2024-08-16

**Authors:** Suelen Fernandes Padilha, Adriana Mércia Guaratini Ibelli, Jane Oliveira Peixoto, Maurício Egídio Cantão, Gabriel Costa Monteiro Moreira, Lana Teixeira Fernandes, Fernando Castro Tavernari, Marcos Antônio Zanella Morés, Ana Paula Almeida Bastos, Laila Talarico Dias, Rodrigo Almeida Teixeira, Mônica Corrêa Ledur

**Affiliations:** 1 Departamento de Zootecnia, Programa de Pós-Graduação em Zootecnia, Universidade Federal do Paraná, Curitiba 80035-050, PR, Brazil; suelenfpadilha7@gmail.com (S.F.P.); lailatalarico@gmail.com (L.T.D.); rteixeiraufpr@gmail.com (R.A.T.); 2 Embrapa Suínos e Aves, Concórdia 89715-899, SC, Brazil; jane.peixoto@embrapa.br (J.O.P.); mauricio.cantao@embrapa.br (M.E.C.); lanatf@yahoo.fr (L.T.F.); fernando.tavernari@embrapa.br (F.C.T.); marcos.mores@embrapa.br (M.A.Z.M.); ana.bastos@embrapa.br (A.P.A.B.); 3 Programa de Pós-Graduação em Ciências Veterinárias, Universidade Estadual do Centro Oeste, Guarapuava 85040-167, PR, Brazil; 4 UVSQ, INRAE, BREED, Université Paris-Saclay, 78350 Jouy-en-Josas, France; gabriel.costa-monteiro-moreira@inrae.fr; 5 Programa de Pós-Graduação em Zootecnia, Universidade do Estado de Santa Catarina, UDESC-Oeste, Chapecó 89815-630, SC, Brazil

**Keywords:** gene expression, pectoral myopathy, RNA-Seq, transcriptome

## Abstract

**Simple Summary:**

White striping (WS) is a condition frequently observed in poultry farming, which reduces consumer acceptability and causes economic losses for the industry. Although common, the development of WS during the animal’s growth remains unclear. Therefore, the objective of this study was to identify genes involved in the occurrence of WS in broiler chickens at 35 days of age and through which processes they act. For this, breast samples were collected from normal and WS-affected chickens, and complete RNA sequencing was performed. Thirty genes showing an altered expression were identified, of which 23 were more expressed and 7 were less expressed in affected chickens compared to normal ones. Fourteen of these genes were related to WS for the first time and are associated with muscle development (*CEPBD*, *DUSP8*, *METTL21EP*, *NELL2*, *UBE3D)*, lipid metabolism (*PDK4*, *DDIT4*, *FKBP5*, *DGAT2*, *LIPG*, *TDH*, and *RGCC*), and collagen (*COL4A5* and *COL4A6*). Genes related to muscle, as well as cellular processes, are possibly involved with the initial phase of WS development. Genes linked to lipid metabolism and collagen may be more related to the progression of this condition.

**Abstract:**

White striping (WS) is a myopathy characterized by the appearance of white stripes parallel to the muscle fibers in the breast of broiler chickens, composed of adipose and connective tissues. This condition causes economic losses and, although common, its etiology remains poorly understood. Hence, the objective was to identify genes and biological mechanisms involved in the early stages of WS using a paternal broiler line that grows slightly slower than commercial ones, at 35 days of age, through the RNA sequencing of the pectoralis major muscle. Thirty genes were differentially expressed between normal and WS-affected chickens, with 23 upregulated and 7 downregulated in the affected broilers. Of these, 14 genes are novel candidates for WS and are implicated in biological processes related to muscle development (*CEPBD*, *DUSP8*, *METTL21EP*, *NELL2*, and *UBE3D*), lipid metabolism (*PDK4*, *DDIT4*, *FKBP5*, *DGAT2*, *LIPG*, *TDH*, and *RGCC*), and collagen (*COL4A5* and *COL4A6*). Genes related to changes in muscle fiber type and the processes of apoptosis, autophagy, proliferation, and differentiation are possibly involved with the initial stage of WS development. In contrast, the genes linked to lipid metabolism and collagen may have their expression altered due to the progression of the myopathy.

## 1. Introduction

White striping (WS) myopathy is characterized by the appearance of white stripes, composed of adipose and connective tissue, that run parallel to the muscle fibers, predominantly in the pectoralis major muscle and less frequently in the thigh muscles [1,2]. This condition predominantly affects birds with high growth rates, greater breast yields, and heavier weights at slaughter [3,4]. This myopathy is undesirable as it impacts the visual appearance, nutritional value, and processing yield of the meat, as well as reduces consumer acceptability and leads to economic losses [1]. WS is considered one of the most common myopathies in poultry farming, with an incidence rate of 10 to 12% on commercial broiler farms [5,6].

Although it is a common condition, the etiology of WS remains poorly understood. It has been determined that the expression of this pathology is partially genetic, with heritability estimates ranging from low to high (0.19 to 0.65) [7,8,9]. These studies have also reported genetic correlation between WS and other traits, with estimates ranging from 0.08 to 0.33 for body weight and from 0.03 to 0.68 for breast yield [7,9]. Genome-wide association studies (GWASs) [10,11,12] have identified several candidate genes for WS and indicated that it is a polygenic trait with no specific genomic region contributing a large portion to the overall genetic variance [13]. However, candidate genes in chromosomes 1, 5, 11, 17, and 20 have been identified [10,14].

Additionally, it has been suggested that the occurrence of WS is related to processes such as hypoxia, oxidative stress, immune system activation, angiogenesis, disruptions in calcium pathways, endoplasmic reticulum (ER) stress, apoptosis, collagen metabolism, autophagy, insulin signaling, circulatory system development, cell response to stimulus, and cytokine production, among others [12,13,15,16,17,18]. However, conclusive evidence regarding the etiology of WS and its temporal progression remains elusive, indicating the need for studies to better understand the development of this myopathy over time [18].

Most research has concentrated on hybrid animals from commercial lines with high breast yields. Moreover, few studies have been conducted with animals younger than 42 days, which could further elucidate the development of this myopathy in broiler chickens of different lines, particularly in pure lines undergoing intense selection. Therefore, the objective of this study was to identify genes and biological mechanisms involved in the occurrence of WS in a Brazilian paternal broiler line at 35 days of age.

## 2. Materials and Methods

### 2.1. Animals and Sample Collection

Male broilers from a paternal broiler line called TT, developed by Embrapa Swine and Poultry National Research Center, were raised until 35 days of age on the experimental farm of the same institution, located in Concórdia, Santa Catarina State, Brazil. For this line, selection emphasis was on improving body weight, feed conversion, carcass and cuts yield, viability, fertility, and hatchability [19]. The TT line has been used as a reference population for genomic studies in chickens, and for more details on the selection and structure of this broiler line, see Marchesi et al. [20].

At the hatchery, the chicks were vaccinated against fowlpox and Marek’s disease. The management conditions adhered to line-specific guidelines, providing water and feed (formulated according to Rostagno et al. [21]) ad libitum. At 35 days of age, the chickens were euthanized by cervical dislocation, following the ethical standards set by the Ethics Committee on Animal Use at Embrapa Swine and Poultry (approval no. 008/2019) and in line with international animal welfare guidelines.

After slaughter, the animals were necropsied, and the breast muscles were visually evaluated for the presence of WS according to the classification by Kuttappan et al. [22]. All affected animals exhibited a mild degree of myopathy. Subsequently, approximately 1 g samples were collected from the cranial region of the pectoralis major muscle (PMM) of eight animals: four samples from animals displaying mild WS (affected) and four from normal broilers (controls), with no evidence of WS or other myopathic changes. These samples were immediately frozen in liquid nitrogen and stored at −80 °C for gene expression analysis. Samples for histological analysis were collected from the opposite side of the breast and preserved in 4% paraformaldehyde.

### 2.2. Histopathological Analysis

The samples were sectioned into 5 m sections, dehydrated in alcohol, cleared in xylol, and embedded in paraffin. The tissues were then sectioned into 3 μm sections, mounted on slides, and stained with hematoxylin and eosin for the morphological evaluation and identification of myopathic lesions.

### 2.3. RNA Extraction, Preparation, and Sequencing of RNA-seq Libraries

Approximately 100 mg of PMM tissue was homogenized in a mortar containing liquid nitrogen and combined with 1 mL of Trizol reagent (Invitrogen, Waltham, MA, USA) for total RNA extraction, followed by purification using the RNeasy mini kit (Qiagen, Hilden, Germany). RNA purity and concentration were measured using a Biodrop spectrophotometer (Biochrom, St. Albans, UK), and integrity was assessed using an Agilent 2100 Bioanalyzer (Agilent Technologies, Santa Clara, CA, USA). Only samples with RIN > 8.0 were used to prepare libraries using the Illumina Stranded mRNA Prep kit (Illumina, San Diego, CA, USA) with 2 μg of total RNA following the manufacturer’s protocol. Library size was assessed on an Agilent 2100 Bioanalyzer (Agilent Technologies, Santa Clara, CA, USA), and quantified by qPCR. Paired-end sequencing (2 × 100 bp) was performed on an Illumina NextSeq 2000 (Illumina, San Diego, CA, USA), at the NGS Soluções Genômicas facility (Piracicaba, SP, Brazil). The sequence files obtained were in fastq format.

### 2.4. RNA-seq Data Analysis

Quality control was conducted using Trimmomatic software v0.39 [23], which removed short sequences (<70 bp), low-quality sequences (QPhred ≤20), and adapter sequences. After quality control, the sequences were mapped against the chicken reference genome (GRCg7b, Ensembl release 111) using STAR software v. 2.7.11a [24], and reads were counted with HTseq-count [25]. Differential expression analysis between normal and WS-affected animals was performed using the edgeR package [26] from R v 4.3.2 [27], and gene hierarchies were visualized in heatmap form. Genes were considered differentially expressed (DE) if they had a false discovery rate (FDR) < 0.05 after correction for multiple testing using the Benjamani–Hochberg (BH) method [28]. DE genes with negative and positive log2 fold change (Log2FC) were considered downregulated and upregulated, respectively, in WS-affected chickens compared to normal chickens.

### 2.5. Functional Annotation and Gene Interaction Networks

Gene ontology analysis was conducted using the annotation packages org.Gg.eg.db [29] and AnnotationDbi [30] from Bioconductor 3.18 [31], utilizing information from *Gallus gallus* (GRCg7b). The functional annotation of the DE genes and the enriched biological processes (BPs) were identified through the clusterProfiler package [32] from Bioconductor, using the MSigDB database [33] with information pertaining to *Gallus gallus* (GRCg7b). Gene interaction analysis was performed with gene expression data from the DE genes using the STRING database [34] with annotations for *Homo sapiens* to predict genetic interactions based on co-expression and co-localization. Finally, genes newly identified as related to WS were termed “novel candidate genes for WS.” A search based on the initial and final positions of each of these genes was conducted to determine whether they were located in regions of quantitative trait loci (QTLs) previously identified in the chicken genome using the chickenQTLdb [35].

### 2.6. qPCR Confirmation Analysis

To confirm the RNA-Seq results, 20 samples (10 normal and 10 affected) were collected and underwent total RNA extraction with the Trizol standard protocol (Invitrogen, Waltham, MA, USA). The total RNA was submitted to first-strand cDNA synthesis using SuperScript III First-Strand Synthesis SuperMix (Invitrogen, Waltham, MA, USA), following the manufacturer’s instructions.

Five candidate genes widely used as reference were chosen to be evaluated according to their stability to be used as normalizers in the gene expression analysis from the *pectoral major* muscle tissue: *HMBS* (Hydroxymethylbilane Synthase) [36], *MRPS30* (Mitochondrial Ribosomal Protein S30) [37], *RPL30* (Ribossomal Protein L30) [38], *RPL4* (Ribosomal Protein L4) [38], and *RPL5* (Ribosomal Protein L5) [37] (Supplementary File S1, Table S1). The qPCR reactions were performed with QuantStudio 6 Real-Time PCR equipment (Life Technologies, Carlsbad, CA, USA) using the following protocol: 1X GoTaq qPCR Master Mix (Promega, WI, USA), 0.133 uM forward primer, 0.133 uM reverse primer, cDNA in 1:10 dilution, and ultra-pure water (Promega, WI, USA) up to 15 μL of final volume. All reactions were performed in duplicate, using negative controls to detect possible contamination. The cycling conditions were 95 °C for 2 min, 40 cycles of 15 s at 95 °C and 30 s at 60 °C, and a melting curve analysis step was added following the manufacturer protocol. The cycle threshold (Ct), standard deviation (SD), and coefficient of variation (CV) were obtained for each sample. Primer’s efficiencies were obtained with Web-based LinRegPCR [39,40,41,42]. Ct means were used to assess the gene stability performed with the automated pipeline endoGenes [43]. This pipeline uses the three most known programs to evaluate gene stability, geNorm, NormFinder, and BestKeeper, generating a final ranking using the RankAggreg package with the most stable genes. *HMBS* and *RPL30* were selected as the most stable genes found for pectoral major muscle tissue (Supplementary File S1: Figure S1).

Nine candidate genes were chosen for qPCR confirmation analysis. The primers for the genes: Pyruvate dehydrogenase kinase 4 (*PDK4*), FK506 binding protein 5 (*FKBP5*), CCAAT enhancer binding protein delta (*CEBPD*), Diacylglycerol O-acyltransferase 2 (*DGAT2*), regulator of cell cycle (*RGCC*), cholinergic receptor nicotinic gamma subunit (*CHRNG*), carbonic anhydrase 3A (*CA3A*), and uncoupling protein 3 (*UCP3*) were designed using the PRIMER BLAST program [44] with sequences downloaded from the NCBI [45] and Ensembl 111 [46] databases (Supplementary File S1: Table S2). Primer sequence for four and a half LIM domains 1 (*FHL1*) gene was obtained from a previous study [12] (Supplementary File S1: Table S2). For the qPCR reactions, 1X of GoTaq qPCR Master Mix (Madison, WI, USA), 0.133 uM of forward primer and 0.133 uM reverse primer, cDNA in 1:10 dilution, and ultra-pure water (Promega, WI, USA) were used. qPCR reactions were performed in duplicate in 15 µL of final volume. Subsequently, qPCR reactions were submitted to the QuantStudio 6 Real-Time PCR equipment (Applied Biosystems, Foster City, CA, USA). The reaction conditions to assess the specificities of the amplification were as follows: 95 °C for 2 min, 40 cycles for 15 s at 95 °C, and 30 s at 60 °C. The primer efficiencies were obtained with Web-based LinRegPCR [39,40,41,42].

The expression level of target genes was determined using Ct values, and changes in gene expression were calculated with the Pfaffl method, considering primer’s efficiency [47], using the geometric mean of the endogenous genes *HMBS* and *RPL30*. A pairwise correlation using the ggpubr package from the R v. 4.3.2 environment [27] was performed to compare and confirm the expression levels between the RNA-Seq (log2 fold-change, log2FC) and qPCR (fold change, FC) data, according to Zhang et al. [48].

## 3. Results

**Figure 1:**
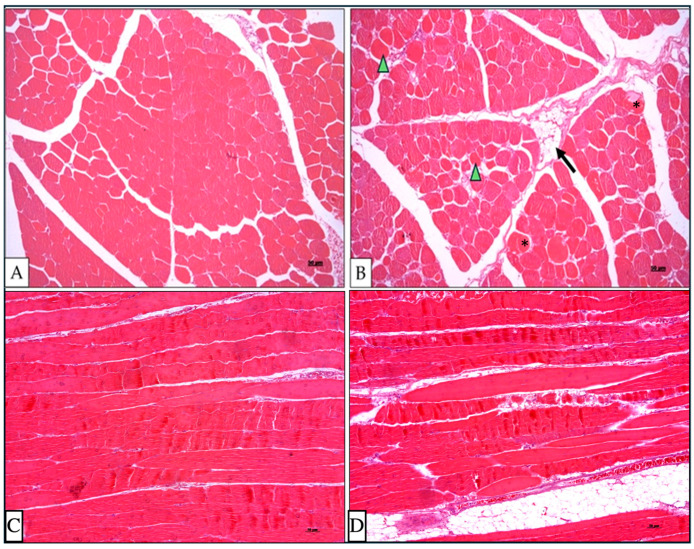
Histopathological analysis of 35-day-old chicken breasts showing microscopic features of normal (**A**,**C**) and white striping (**B**,**D**) muscles. Presence of hypereosinophilic fibers (arrowhead), grade of muscular degeneration (*) and adipose infiltration (arrow)—hematoxylin and eosin stain.

**Figure 2:**
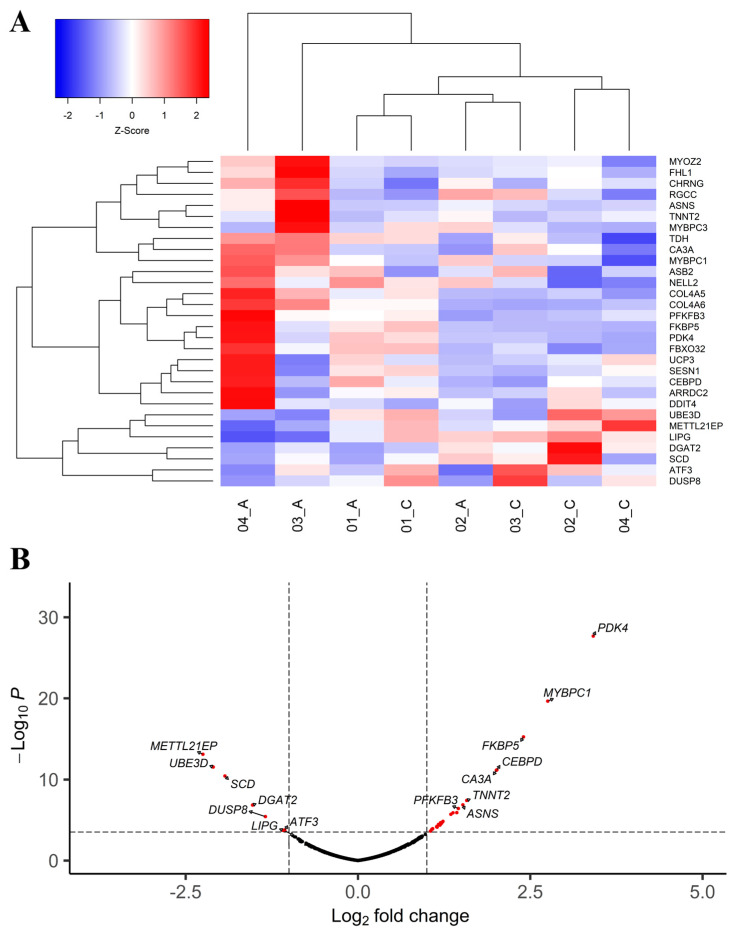
(**A**) A heatmap showing a hierarchical clustering of the genes and samples of the DE genes between WS-affected and normal broilers. Genes are presented in the rows, and samples, in the columns. Downregulated genes in the affected group are shown in blue, and upregulated genes, in red. (**B**) A volcano plot showing the main differentially expressed genes in WS-affected broilers (-Log_10_ *p* < 3.699 is equal to FDR < 0.05).

The histopathological analysis and the macroscopic evaluation revealed two types of samples: (1) normal muscles, showing organized muscle fibers with homogeneous size and coloration (Figure 1A,C), and (2) samples affected by WS, characterized by fibers of varying sizes, mild presence of hypereosinophilic, rounded fibers with hyaline degeneration, and infiltration of adipose tissue within the connective tissue (lipidosis) (Figure 1B,D).

The sequencing of PMM samples generated approximately 18.2 million reads per sample (minimum: 15.9 million; maximum: 20.6 million). After quality control, about 17.2 million reads/sample remained, of which 88.1% were mapped to the chicken reference genome (GRCg7b, Ensembl release 111), and 75.5% of these reads were mapped to gene regions (Supplementary File S1: Table S3).

The hierarchical cluster analysis of DE genes showed that although distinct expression patterns between normal and affected chickens were observed for some genes, they were not homogeneous across all affected samples (Figure 2A). This profile was primarily influenced by the mild degree of WS present in the affected animals, resulting in less stark contrasts compared to normal broilers.

A total of 7434 genes were expressed in the PMM transcriptome (Supplementary File S1, Table S4), among which 30 were DE between normal and WS-affected chickens (Table 1). Of these DE genes, 23 were upregulated and 7 were downregulated in the affected group compared to the normal counterpart (Figure 2B; Table 1). Fourteen novel candidate genes for WS were identified: *PDK4*, *DDIT4*, *FKBP5*, *DGAT2*, *LIPG*, *TDH*, *RGCC*, *CEPBD*, *DUSP8*, *METTL21EP*, *NELL2*, *UBE3D*, *COL4A5*, and *COL4A6* (Table 1).

**Figure 3:**
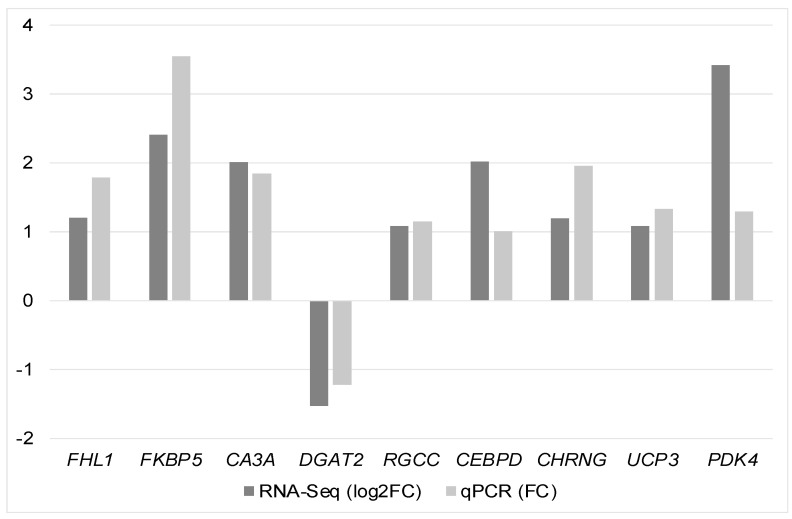
Expression levels of RNA-Seq (log2FC) and qPCR (FC).

From the nine genes selected for qPCR confirmation, it was possible to verify a high correlation between the expression levels of the RNA-Seq and the qPCR analysis (Figure 3) (r = 0.71), evincing consistent results from the RNA-Seq obtained in this study; therefore, the qPCR analysis validated the RNA-Seq results (Supplementary File S1: Figure S2).

In the search for positional candidate genes, 13 of the novel candidate genes for WS were found in regions associated with QTLs for the following traits: body weight (including weight gain, average daily gain, carcass weight, conformation score, feed conversion, and feed efficiency), pectoral muscle and PMM (including breast weight, percentage, and width), muscle fiber (including number, diameter, and density), and fat content/percentage (intramuscular and carcass) (Supplementary File S1: Table S5). The *PDK4*, *NELL2*, *DUSP8*, and *COL4A5* genes are notably located in QTL regions associated with many of the aforementioned traits.

**Figure 4:**
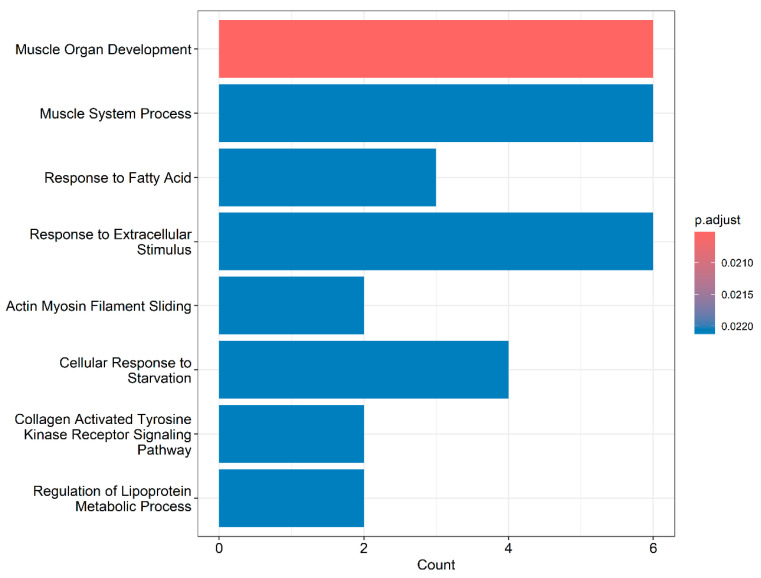
Main biological process enriched from combined differentially expressed genes (up- and downregulated) between WS-affected and normal broilers.

Gene ontology analysis revealed that DE genes were enriched in 518 BPs, with 23 reaching statistical significance (*p* < 0.05; Supplementary File S1: Table S6). The most representative processes are illustrated in Figure 4.

**Figure 5:**
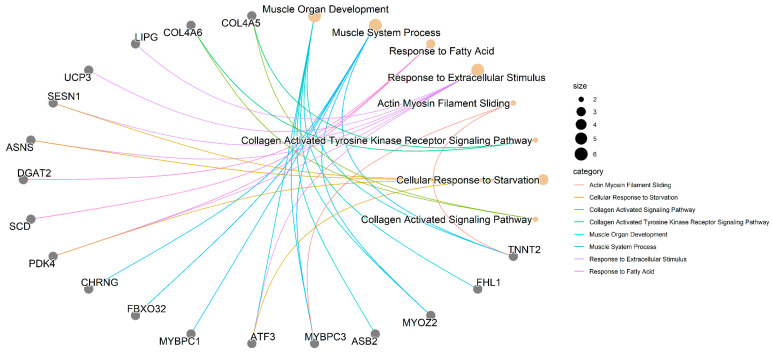
Gene enrichment analysis of differentially expressed genes and biological processes related to WS in broiler chickens. Gray circles represent DE genes in WS-affected compared to normal broilers. Flesh-colored circles represent biological processes; their size indicates the number of related genes. Colored lines denote the category of the biological process.

In Figure 5, it is possible to identify which genes are associated with the most relevant BPs. Some genes are shown to participate in more than one BP, and these can be categorized into three major groups: (1) muscle-related genes (*CHRNG*, *FBXO32*, *MYBPC1*, *ATF3*, *MYBPC3*, *ASB2*, *MYOZ2*, *FHL1*, and *TNNT2*); (2) genes involved in lipid metabolism and cellular processes (*LIPG*, *UCP3*, *SESN1*, *ASNS*, *DGAT2*, *SCD*, *PDK4*, and *ATF3*); and (3) genes associated with collagen (*COL4A5* and *COL4A6*).

The gene network (Figure 5), constructed with the expression data from DE genes, demonstrates interactions among most of the candidate genes, indicating functional associations that may contribute to the manifestation of WS. Additionally, there is a notable connection between this network and significant BPs, as the interacting genes (Figure 5) also participate in the same BPs (Figure 4), which reinforces the importance and functional relationships of these genes.

**Figure 6:**
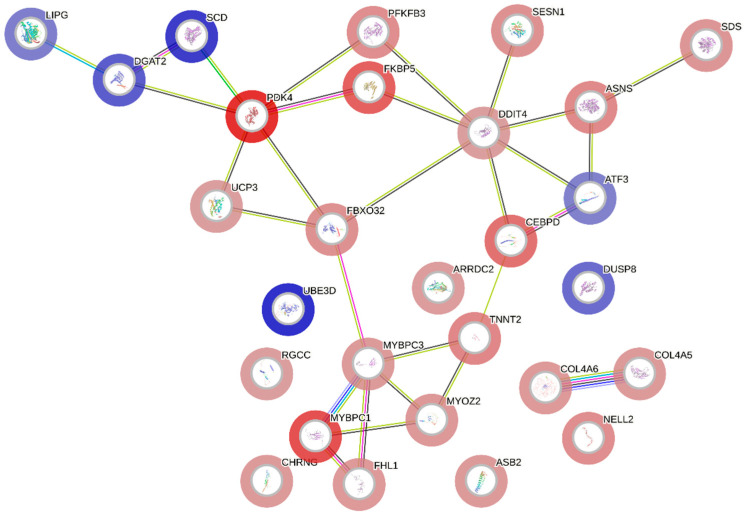
Gene network of differentially expressed genes between WS-affected and normal broilers. Upregulated genes in the affected group are shown in red, and downregulated genes, in blue. Lines indicate gene interactions.

The interaction among genes from the three large aforementioned groups (related to muscle development, lipid metabolism and cellular processes, and collagen) are shown in Figure 6. The *PDK4* and *DDIT4* genes, which were upregulated in the WS-affected group (Table 1), were the main interactors within the network (Figure 5), forming two clusters related to lipid metabolism and cellular processes. Another important cluster was formed by muscle-related genes, featuring *MYBPC1*, *MYBPC3*, *FHL1*, *MYOZ2*, *TNNT2*, *CEBPD*, and *FBXO32*, and a separate cluster of two genes related to collagen processes (*COL4A5* and *COL4A6*), all upregulated in WS-affected broilers.

## 4. Discussion

In this study, we investigated the global gene expression profile of the pectoralis major muscle (PMM) of normal and WS-affected broilers from a Brazilian paternal line at 35 days of age. This line has a slightly lower growth rate compared to other commercial lines, which may contribute to a reduced incidence of WS and milder degrees of the myopathy. Furthermore, the samples were obtained from 35-day-old broilers, while most studies published so far have typically involved older animals. It is interesting to note that there are no gene expression studies on WS with broilers that are at ages less than 42 days old, differing from Wooden breast studies. Therefore, it was possible to investigate the gene expression profile of broilers at an earlier stage of the myopathy development, with the identification of 30 DE genes between WS-affected chickens and their normal counterpart. These genes are implicated in biological processes related to muscle development, lipid metabolism and cellular processes, and collagen. Among the DE genes, fourteen candidate genes for WS were identified for the first time: *CEPBD*, *METTL21EP*, *DUSP8, NELL2*, *UBE3D*, *PDK4*, *DDIT4*, *FKBP5*, *DGAT2*, *LIPG*, *TDH*, *RGCC*, *COL4A5*, and *COL4A6*. These genes likely emerged, possibly due to the early age and the specific broiler line used, which allowed the evaluation of initial stages of the myopathy. Moreover, these genes are located in regions of QTL associated with traits such as body weight, pectoral muscle and PMM, muscle fiber, and intramuscular and carcass fat content/percentage. These findings highlight the importance of the biological pathways involved (muscle development and lipid metabolism) and reinforce the suggested role of these genes in modifying the muscle fiber type, a condition described in the literature as linked to breast myopathies [49], and in the accumulation of fat between muscle fibers, as observed in the histopathological analysis (Figure 1B).

### 4.1. Muscle-Related Genes

The association of genes related to muscle development processes with WS myopathy in broiler chickens has been previously reported [14,18,50]. In our study, muscle-related BPs were significant (Figure 3 and Supplementary File S1: Table S6), involving genes such as *MYBPC1*, *MYBPC3*, *MYOZ2*, *FHL1*, and *TNNT2*. These genes interact with each other and with *CEBPD*, *ATF3*, and *FBXO32* (Figure 5), and, along with *CHRNG* and *ASB2*, participate in these BPs (Supplementary File S1: Table S6). Except for *ATF3*, which was downregulated, all other genes in this group were upregulated in broilers affected with WS.

The overexpression of genes such as *MYBPC1*, *MYBPC3*, *MYOZ2*, and *TNNT2* has already been linked to muscle growth and the development of pectoral myopathies, including WS [12,49,51,52,53]. The myosin-binding protein C (MyBP-C) family is a group of sarcomeric proteins that are crucial for the structure and function of striated muscle. This family is composed of three isoforms, cardiac, slow skeletal, and fast skeletal, which are encoded by *MYBPC3*, *MYBPC1*, and *MYBPC2*, respectively [54]. The upregulation of these genes may shift muscle fibers from fast (glycolytic) to slow (oxidative), altering muscle structure [49,51,52,53] and affecting the contractile, metabolic, and biochemical properties of the muscle [55]. It has been shown that higher levels of the *MYOZ2* gene can decrease the expression of calcineurin, which is involved in the development and differentiation of skeletal muscle as well as in controlling muscle fiber type [56,57]. The *TNNT2* gene also regulates calcium for the contraction of striated muscles [58]. The overexpression of this gene reduces the calcium load in muscles [59], and disruptions in calcium pathways can trigger WS in chickens [18].

Another gene that could influence muscle fiber type in response to metabolic imbalances is *FHL1*. The upregulation of this gene enhances the expression of the slow oxidative fiber type [60] and has also been associated with WS [12,61] and Wooden Breast (WB) in commercial broiler chickens [62]. The overexpression of the *CA3A* gene (Figure 2 and Table 1) supports the hypothesis of a shift in muscle fiber type in WS-affected chickens, as *CA3A* is a marker for slow-twitch fiber types [63]. *CA3A* can be regulated by hypoxia-inducible factor 1 (HIF-1) and has been reported to be upregulated in chickens affected with WB in a pure line at 47 days [49] and with WS in the Cobb line at 42 and 56 days of age [12,61]. Hence, the upregulation of *CA3A* in WS-affected chickens could indicate a hypoxic condition that may have led to tissue damage, resulting in transitioning muscle fibers to a slow (oxidative) type, thereby increasing *CA3A* expression.

*METTL21EP* was identified in the present study as a novel candidate gene for WS, possibly due to the early age and the broiler line used, and its downregulation might also be a consequence of the shift in muscle fiber type. Originating as a pseudogene from *METTL21E* [64], which is predominant in type IIb myofibers, its reduced expression can lead to decreased myofiber size [65]. Thus, the reduced expression of *METTL21EP* in WS-affected chickens likely reflects a shift to predominantly slow (type I, oxidative) muscle fibers. Moreover, this gene was recently reported as DE in chickens with WB at 47 days [66]

The expression of the *DUSP8* gene can be altered in response to oxidative stress [67], and its inhibition shifts skeletal muscle fibers to an oxidative type [68]. *DUSP8* is also located in a region of QTLs associated with traits such as weight gain, carcass weight, conformation, and breast weight and yield, as well as muscle fiber diameter and density (Supplementary File S1: Table S5), highlighting its potential role in the onset of WS. The inhibition of *DUSP8* promotes autophagy, while its increased expression may inhibit cell proliferation and induce apoptosis [69,70]. Therefore, the observed downregulation of *DUSP8* in WS-affected chickens, described for the first time in this study, could be a response to oxidative stress, promoting the autophagy of damaged muscle fibers and proliferative processes typical of oxidative (slow) fibers. This response could enhance lipid oxidation, leading to fat accumulation in the muscles, a hallmark of the WS (Figure 1B). Additionally, in this study, the biological processes related to lipid metabolism were significant (Figure 3).

Further discussing muscle regulation and functioning, additional genes—*FBXO32*, *CEBPD*, *ASB2*, and *CHRNG*—have also been linked to apoptosis and differentiation, processes previously associated with the development of WS [11,50]. The *FBXO32* gene is a key mediator of apoptosis following ischemia injury [71], and apoptosis can lead to tissue damage that requires the activation of repair mechanisms [12]. However, the upregulation of this gene suppresses differentiation and inhibits myotube formation. As this process is essential for repair after injury, it is suggested that the overexpression of *FBXO32* adversely impacts the regeneration of skeletal muscle [72]. In addition, Marchesi et al. [12] observed the downregulation of the *FBXO32* gene in the breast muscles of Cobb chickens with WS at 42 days. In contrast, in the current study involving the TT line chickens with 35 days of age, the *FBXO32* gene was found to be upregulated. The opposite expression levels of this gene observed in both studies may be attributed to the line used, the progression of the myopathy and the degree of fiber regeneration. Moreover, Li et al. [73] also reported a difference in the expression of this gene between pure breeds and a commercial line of broiler chickens.

In our study, the *CEBPD* gene was identified as a novel candidate gene for WS. Although it is not directly linked to skeletal muscle BP (Supplementary File S1: Table S6), it is located in a QTL region associated with breast muscle weight (Supplementary File S1: Table S6). This gene encodes a protein involved in various BPs, including cell differentiation, proliferation, apoptosis, and immune response [74,75]. *CEBPD* is also highly expressed under hypoxic conditions [76] and plays a crucial role in mediating inflammatory responses [77]. Thus, it is hypothesized that WS-affected broilers experienced muscular hypoxia, leading to the overexpression of the *CEBPD* gene. Nonetheless, the specific mechanisms through which this gene contributes to myopathy development need further elucidation.

On the other hand, although the *ASB2* and *CHRNG* genes do not interact with other muscle-related genes, they are involved in BPs related to muscle processes (Figure 4; Supplementary File S1: Table S5). The *ASB2* gene regulates myogenic differentiation through the ubiquitination and degradation of filamin B (FLNb) [78], while the *CHRNG* gene influences myotube differentiation [79]. Therefore, we suggest that in WS-affected animals, both genes (*ASB2* and *CHRNG*), which were upregulated, enhance myogenic differentiation and favor the regeneration of tissue damaged by the possible hypoxic conditions.

Some genes, such as *NELL2* and *ARRDC2* (upregulated), as well as *UBE3D* (downregulated), are not involved in muscle-related BPs (Supplementary File S1: Table S6). However, this study is the first to identify *NELL2* and *UBE3D* as candidate genes for WS. *NELL2* is found in the QTL region associated with body weight, carcass weight, breast weight, intramuscular fat content, and muscle fiber (Supplementary File S1: Table S5). It is known that *NELL2* overexpression may promote cell proliferation and inhibit apoptosis [80]. *ARRDC2* expression increases following catabolic stimuli and might be involved in autophagy activation [81]. Hence, it is suggested that the upregulation of *ARRDC2* in WS-affected broilers was a response to muscle injuries from apoptotic processes and potential oxidative stress. The upregulation of *NELL2* is thought to promote the proliferation and differentiation of muscle cells, aiding in the regeneration of damaged tissue. Finally, although the *UBE3D* gene has been minimally studied, it is located in QTL regions associated with body weight (Supplementary File S1: Table S5). Some reports indicate that its low expression in zebrafish embryos is linked to oxidative damage and inflammatory responses, and that inhibiting this gene can lead to apoptosis and retinal degeneration [82]. Therefore, it is assumed that the downregulation of this gene in the PMM of chickens could have caused oxidative damage and inflammatory reactions, inducing apoptosis and muscle fiber degeneration, which are hallmark histopathological features of WS.

Changes in the expression profiles of genes associated with muscular processes may indicate alterations that are potentially involved in the initial stage of WS development. The PMM is predominantly composed of fast-twitch-type IIb glycolytic muscle fibers [83]. Nonetheless, several factors such as activity rate, mechanical stress, hormones, aging, responses to muscle damage, and/or changes in muscle oxygenation can lead to fiber-type switching [84]. In this way, the upregulation of the *MYBPC1*, *MYBPC3*, *MYOZ2*, *TNNT2*, *FHL1*, and *CA3A* genes, along with the downregulation of *METTL21EP* and *DUSP8*, which are associated with changes in muscle fiber type, are important findings for understanding the alterations in the muscle tissue affected by WS. This study is the first to identify genes associated with muscle fiber type changes in tissues exclusively affected by WS.

### 4.2. Genes Related to Lipid Metabolism and Cellular Processes

The genetic selection applied to improve broiler chickens has led to reduced muscle oxygenation (hypoxia), which, combined with oxidative stress, contributes to disorders in the oxidation of fatty acids and the tricarboxylic acid cycle, resulting in muscle problems [15]. Significant BPs related to lipid metabolism and cellular processes were identified in this study (Figure 3 and Supplementary File S1: Table S6). These processes involve *PDK4* and *DDIT4*, novel candidate genes for WS, which interact with several other genes (*UCP3*, *PFKFB3*, *FKBP5*, *SCD*, *DGAT2*, *LIPG*, *SESN1*, *ASNS*, and *ATF3*) implicated in these BPs (Figure 4 and Figure 5). With the exception of *FKBP5*, all have been linked to breast myopathies [12,52,85]. These findings support the hypotheses of Lake and Abasht [86] and Pizzol et al. [17], who suggested that WS may be caused by dysregulation in glucose and lipid metabolism. Additionally, cellular processes such as autophagy, apoptosis, and differentiation have also been linked to WS [11,17,18,50] and are likely activated in response to the onset of the myopathy.

Several genes identified in the current study are regulated by hypoxic conditions, e.g., *PDK4*, *DDIT4*, *PFKFB3*, *SESN1*, and *CEBPD*. Changes in muscle oxygenation can induce fiber-type switching [84] and activate various cellular signaling pathways that control metabolism. These pathways stimulate the expression of the *PDK4* gene via the nuclear receptor ERRγ [87,88]. In our study, *PDK4* was found to be more expressed in WS-affected chickens, enriched in the BP for fatty acid response (Figure 4), and located in QTL regions for intramuscular fat percentage (Supplementary File S1: Table S5). The increased expression of this gene suggests the enhanced oxidation of fatty acids [89]. Given that WS-affected chickens exhibit high lipid content in the pectoral muscle [90], it is posited that the excessive oxidation of fatty acids could have led to mitochondrial overload and incomplete oxidation, resulting in the intramuscular accumulation of metabolites derived from fatty acids [91]. Moreover, as oxygen is necessary for lipid oxidation, the likely hypoxic condition could have led to incomplete oxidation and intensified the accumulation of fatty acids in the muscle, as evidenced by histopathological analysis (Figure 1B).

The *UCP3* gene interacts with *PDK4* (Figure 5), and, in some way, the overexpression of *PDK4* accounts for the observed increase in *UCP3* expression. This upregulation decreases mitochondrial membrane potential, enhances the ATP/ADP ratio, and favors fatty acid oxidation over glucose utilization [92,93]. *UCP3* primarily facilitates the transport of fatty acid anions from muscle mitochondria, protecting against the detrimental effects of high fatty acid levels [94]. This gene was found to be downregulated in Ross chickens exhibiting severe levels of WS [11]. However, unlike in our study, the comparison was between chickens with severe and mild WS.

Additionally, the *SCD* and *DGAT2* genes also interact with *PDK4* (Figure 5), with *DGAT2* implicated in the BP related to fatty acid response (Figure 4). Nevertheless, these genes were downregulated in WS-affected chickens (Figure 2 and Table 1). *SCD* inhibition enhances β-oxidation in muscles [95], while *DGAT2* inhibition in skeletal myotubes reduces glucose uptake and the partitioning of fatty acids into triacylglycerols (TAGs) [96,97]. Thus, *DGAT2* downregulation may reduce TAG formation and elevate harmful fatty acid levels in muscle tissue (Figure 1B), leading to lipotoxicity and the development of pectoral myopathy [85]. Similarly, *LIPG* downregulation disrupts lipid metabolism, potentially triggering pectoral myopathy alongside other lipid-related genes [52]. Both *DGAT2* and *LIPG* have been previously associated with WB pectoral myopathy [52,85], but are linked to WS for the first time in the present study.

The *TDH* and *RGCC* genes were upregulated in the PMM of WS-affected chickens (Figure 2; Table 1) and, although not involved in the BPs identified in this study (Figure 4), were related to WS for the first time, establishing them as key functional candidates for myopathy development. The *TDH* gene regulates fatty acid degradation, biosynthesis, and ketobody formation [98] and may influence the development of chicken breast muscle fibers [99]. Positioned in QTL for carcass fat content (Supplementary File S1: Table S5), the *RGCC* gene regulates cell differentiation [100], and when upregulated, *RGCC* promotes fatty acid oxidation [101,102], contributing to metabolic imbalance and lipid accumulation in muscle (Figure 1B), which is indicative of WS.

In addition to activating various metabolic control pathways, hypoxic conditions induce the expression of *DDIT4*, whose overexpression can promote apoptosis [103] and inhibit mTOR signaling [104], limiting the autophagy [104,105]. Therefore, the upregulation of *DDIT4* in WS-affected chickens, now identified as a novel candidate gene, could suppress the mTOR pathway. This suppression may enhance apoptosis and autophagy processes to mitigate damage from hypoxia and oxidative stress, serving as an important mechanism for degrading damaged cells in pectoral muscles affected by WS [106].

The *PFKFB3* and *FKBP5* genes interact with both *PDK4* and *DDIT4* (Figure 5). *FKBP5* has emerged as a novel candidate gene for WS. During hypoxic conditions, the hypoxia-inducible factor 1 (HIF-1) complex activates *PFKFB3*, promoting its expression [107]. The overexpression of *PFKFB3* increases cell proliferation [108] and modulates autophagy [109]. Although *FKBP5* does not participate in lipid metabolism or cellular processes (Figure 4 and Supplementary File S1: Table S6), it can enhance autophagy [110] and trigger p53-dependent apoptosis [111]. Furthermore, *FKBP5* upregulation increases muscle cell differentiation and tissue regeneration [112]. Thus, the likely hypoxic environment in PMM affected by WS could stimulate increased expression of both *PFKFB3* and *FKBP5*, which may lead to the apoptosis and autophagy of injured cells while enhancing cell proliferation and differentiation, aiding in tissue regeneration.

In addition to the *PFKFB3* and *FKBP5* genes, *DDIT4* formed a cluster including the *SESN1*, *ASNS*, and *ATF3* genes (Figure 5). Sestrin family genes, such as *SESN1*, are typically induced under conditions of DNA damage, hypoxia, and oxidative stress [113,114]. The overexpression of *SESN1* in chicken skeletal muscle cells stimulates cell proliferation and myoblast differentiation [115]. Similarly, the overexpression of *ASNS* promotes proliferation and resistance to oxidative stress in human stem cells [116]. Therefore, we hypothesize that the overexpression of *SESN1* and *ASNS* could promote muscle proliferation and differentiation in order to control oxidative stress and repair the damaged tissue.

The *ATF3* gene was found to be downregulated in an initial stage of WS development (Figure 2; Table 1). Studies have shown that increased *ATF3* expression can reduce β-oxidation rates, while decreased expression may lessen apoptosis and suppress both the production of reactive oxygen species (ROS) and inflammatory responses [117,118,119]. Therefore, the downregulation of *ATF3* in the pectoral muscles of WS-affected chickens might elevate β-oxidation rates. Alongside the other genes discussed, this could deregulate lipid metabolism, favoring lipid accumulation in muscle fibers (observed in Figure 1B) and contributing to the development of myopathy. Furthermore, the downregulation of *ATF3* might reflect an adaptive response to stabilize oxidative stress, apoptosis, and inflammation during the development of the myopathy, thereby aiding in the repair of muscle tissue damage. However, this gene was previously reported to be upregulated in a study from our group comparing the transcriptome of normal and WS-affected commercial broilers at 42 days of age [12]. This discrepancy indicates that the expression of this gene is modulated by the lipid accumulation and tissue regeneration.

### 4.3. Collagen-Related Genes

Research has documented an association between collagen genes and both WS and WB in broiler chickens [14,16,120]. Additionally, WS-affected chicken breast filets exhibit higher collagen content, including type IV collagen [121,122,123]. In our study, collagen-related BPs were significant (Figure 3 and Figure 4 and Supplementary File S1: Table S6), and the *COL4A5* and *COL4A6* genes, found to be upregulated in WS-affected chickens (Figure 2 and Table 1), were identified as novel candidate genes for WS.

Type IV collagen is the primary structural component of the basement membrane, as it forms a supportive network beneath epithelial and endothelial cells and serves as a barrier between tissue compartments [124]. The upregulation of the *COL4A5* and *COL4A6* genes in affected animals could likely result from myofibril necrosis [125], which may lead to collagen accumulation. The accumulation of type IV collagen can induce ER stress, causing alterations in the structure of the extracellular matrix and apoptosis in the pectoral muscles affected by myopathies [120].

Therefore, it is plausible that an imbalance of hypoxia and oxidative stress mechanisms in the pectoralis major muscle may have facilitated the emergence of tissue injuries. For tissue regeneration, apoptosis and autophagy could be induced, along with proliferation and myogenic differentiation. This could result in a transition of muscle fibers from fast (glycolytic) to slow (oxidative) types. The predominance of slow-type fibers could have disrupted metabolism, leading to enhanced fatty acid degradation and/or incomplete oxidation. Excessive or incomplete lipid oxidation may cause lipid accumulation in muscle, a phenomenon partly characteristic of WS myopathy. On the other hand, tissue injuries might also trigger excessive collagen production in an attempt to rebuild the muscle’s basement membrane. This collagen accumulation could induce endoplasmic reticulum stress, further contributing to the development of WS. Further studies with this broiler line at an earlier age could help elucidate the mechanisms activating muscle genes and determine whether there was indeed a state of hypoxia and oxidative stress.

## 5. Conclusions

In the initial stages of WS, observed in the evaluated broiler line at 35 days of age, a set of 30 differentially expressed genes was identified. Among these, *CEBPD*, *METTL21EP*, *DUSP8*, *NELL2*, *UBE3D*, *PDK4*, *DDIT4*, *FKBP5*, *DGAT2*, *LIPG*, *TDH*, *RGCC*, *COL4A5*, and *COL4A6* were associated with this myopathy for the first time. Thirteen of these novel candidates for white striping were located in QTL regions linked to the traits of body weight, carcass and breast weight, muscle fibers, and intramuscular fat content. Moreover, 15 genes were associated with muscle biological processes, including 8 linked to changes in muscle fiber type. These, along with other genes involved in apoptosis, autophagy, proliferation, and differentiation, are highlighted as likely involved in the early stages of WS development. This finding demonstrates the potential shift from glycolytic to oxidative muscle fiber types in WS-affected chickens and suggests mechanisms for lipid accumulation in muscles exhibiting WS. In this sense, eight genes involved in lipid metabolism and two related to collagen were also identified, whose altered expression may be due to the development of the myopathy.

## Figures and Tables

**Table 1:** Differentially expressed genes up- and downregulated in WS-affected broilers, according to FDR (novel genes associated with white striping are shown in bold).

		**Upregulated**			
**Chromosome**	**Associated Gene Name**	**Gene Description**	**Ensembl Gene Id**	**logFC**	**FDR**
2	***PDK4***	pyruvate dehydrogenase kinase 4	ENSGALG00010002741	3.41421	1.571 × 10^−24^
1	*MYBPC1*	myosin binding protein C1	ENSGALG00010011558	2.75445	8.349 × 10^−17^
26	***FKBP5***	FK506 binding protein 5	ENSGALG00010026893	2.40214	1.345 × 10^−12^
2	***CEBPD***	CCAAT enhancer binding protein delta	ENSGALG00010005405	2.01735	7.776 × 10^−9^
2	*CA3A*	carbonic anhydrase 3A	ENSGALG00010013288	2.00895	7.776 × 10^−9^
26	*TNNT2*	troponin T2, cardiac type	ENSGALG00010026743	1.57929	3.182 × 10^−5^
2	*ASNS*	asparagine synthetase (glutamine-hydrolyzing)	ENSGALG00010003382	1.52297	9.686 × 10^−5^
1	*PFKFB3*	6-phosphofructo-2-kinase/fructose-2,6-biphosphatase 3	ENSGALG00010006245	1.45674	0.0002375
1	***NELL2***	neural EGFL like 2	ENSGALG00010013749	1.43497	0.0006832
2	*FBXO32*	F-box protein 32	ENSGALG00010008245	1.37948	0.0006832
3	*SESN1*	sestrin 1	ENSGALG00010014244	1.34848	0.0009661
3	***TDH***	L-treonina desidrogenase	ENSGALG00010011278	1.23466	0.0064443
4	*MYOZ2*	myozenin 2	ENSGALG00010005510	1.21752	0.0089447
4	***COL4A6***	collagen type IV alpha 6 chain	ENSGALG00010015733	1.20722	0.0107838
4	*FHL1*	four and a half LIM domains 1	ENSGALG00010014929	1.20013	0.0088713
9	*CHRNG*	cholinergic receptor nicotinic gamma subunit	ENSGALG00010017878	1.18978	0.0143605
5	*ASB2*	ankyrin repeat and SOCS box containing 2	ENSGALG00010016987	1.17026	0.0122158
4	***COL4A5***	collagen type IV alpha 5 chain	ENSGALG00010015632	1.14888	0.0228397
5	*MYBPC3*	myosin binding protein C3	ENSGALG00010022468	1.14728	0.0190541
28	*ARRDC2*	arrestin domain containing 2	ENSGALG00010027232	1.14249	0.0216413
1	*UCP3*	uncoupling protein 3	ENSGALG00010002543	1.08505	0.0337988
6	***DDIT4***	DNA damage inducible transcript 4	ENSGALG00010021509	1.0787	0.0370386
1	***RGCC***	regulator of cell cycle	ENSGALG00010003192	1.07199	0.0372903
		**Downregulated**			
**Chromosome**	**Associated gene name**	**Gene description**	**Ensembl gene id**	**logFC**	**FDR**
1	***METTL21EP***	methyltransferase like 21E, pseudogene	ENSGALG00010006037	−2.2487	1.40 × 10^−10^
3	***UBE3D***	ubiquitin protein ligase E3D	ENSGALG00010003115	−2.0996	4.26 × 10^−9^
6	*SCD*	stearoyl-CoA desaturase	ENSGALG00010021083	−1.9290	3.38 × 10^−8^
1	***DGAT2***	diacylglycerol O-acyltransferase 2	ENSGALG00010003035	−1.5278	9.69 × 10^−5^
5	***DUSP8***	dual specificity phosphatase 8	ENSGALG00010024637	−1.3427	0.0017
Z	***LIPG***	lipase G, endothelial type	ENSGALG00010009013	-1.0878	0.0466
3	*ATF3*	activating transcription factor 3	ENSGALG00010019307	−1.0621	0.0431

## Data Availability

All data are available in the article.

## Supplementary Materials

The following supporting information can be downloaded at https://www.mdpi.com/article/10.3390/ani14162379/s1: Supplementary File S1: Table S1: Primers for the reference candidate genes evaluated in pectoral major muscle tissue; Supplementary File S1: Figure S1: Overall ranking of reference candidate genes based on the Genorm, Normfinder, and Bestkeeper software ranked using RankAggreg tool; Supplementary File S1: Table S2: Primers for the candidate genes evaluated in pectoral major muscle tissue; Supplementary File S1: Table S3: Number of raw reads, after quality control and mapped reads; Supplementary File S1: Table S4: Genes expressed in the pectoralis major muscle of normal and WS-affect broilers; Supplementary File S1: Figure S2: Pearson correlation (r) obtained between RNA-Seq and qPCR of the nine selected genes. The straight line is the linear regression, the genes are shown by the black dots, and the gray area represents the 95% confidence interval; Supplementary File S1: Table S5: List of QTLs located in the region of novel candidate genes for white striping in broilers; Supplementary File S1: Table S6: Biological processes enriched with differentially expressed genes between white striping-affected and normal broilers.
